# A Review on Porosity Formation in Aluminum-Based Alloys

**DOI:** 10.3390/ma16052047

**Published:** 2023-03-01

**Authors:** Agnes M. Samuel, Ehab Samuel, Victor Songmene, Fawzy H. Samuel

**Affiliations:** 1 Département des Sciences Appliquées, Université du Québec à Chicoutimi, Saguenay, QC G7H 2B1, Canada; 2 Department of Mechanical Engineering, École de Technologie Supérieure (ÉTS), Montréal, QC H3C 1K3, Canada

**Keywords:** porosity, hydrogen, modification, grain refining, solidification rate, statistical modeling

## Abstract

The main objective of this review is to analyze the equations proposed for expressing the effect of various parameters on porosity formation in aluminum-based alloys. These parameters include alloying elements, solidification rate, grain refining, modification, hydrogen content, as well as the applied pressure on porosity formation in such alloys. They are used to establish as precisely as possible a statistical model to describe the resulting porosity characteristics such as the percentage porosity and pore characteristics, as controlled by the chemical composition of the alloy, modification, grain refining, and the casting conditions. The measured parameters of percentage porosity, maximum pore area, average pore area, maximum pore length, and average pore length, which were obtained from statistical analysis, are discussed, and they are supported using optical micrographs, electron microscopic images of fractured tensile bars, as well as radiography. In addition, an analysis of the statistical data is presented. It should be noted that all alloys described were well degassed and filtered prior to casting.

## 1. Introduction

**Figure 1:**
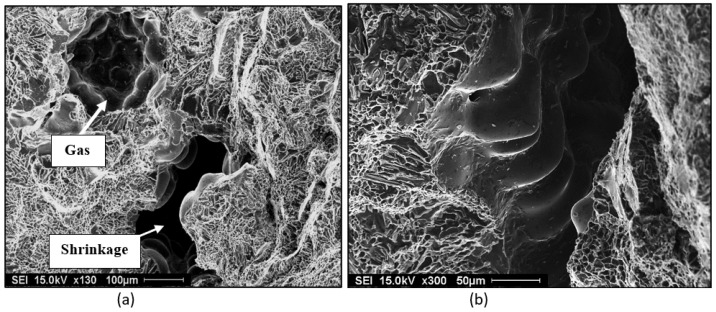
SEM electron images of porosity in 319 alloy (**a**) a mixture of gas and shrinkage, (**b**) a high magnification micrograph of shrinkage reveals dendrites.

The formation of porosity in aluminum is attributable to the combination of two important factors: the dissolution of hydrogen in the metal bath, hydrogen being the only gas with solubility in aluminum, and shrinkage during the solidification of the metal. In addition, melt treatment (modification and grain refining) also affect the porosity formed. Hydrogen is very soluble in liquid metal. When the metal cools, the solubility decreases, and upon solidification, hydrogen is released through the solid/liquid interface. There is then an increase in the concentration of hydrogen in the interdendritic liquid. When the hydrogen concentration becomes higher than the solubility limit in the metal, the germination process begins. Germination is the process by which hydrogen bubbles concentrate in the interdendritic liquid. If their energy is sufficient, they increase in volume, but they remain trapped in the pasty zone. After solidification, the porosity volume increases slightly due to solidification shrinkage [1,2,3,4,5,6,7,8,9,10]. Examples of gas and shrinkage porosity are shown in Figure 1.

The growth and nucleation criterion of a pore is defined by the following inequality formula [11,12,13]:

(1) Pgas ≥P atm+Pmet+Pretrait+Psurface=Pliq+P surface

The *Pgas* term of the inequality formula is responsible for the expansion of the gas bubbles, while the other terms affect the pore growth. By definition, *Pgas* is the pressure inside the pore, *Patm* is the ambient atmospheric pressure, and *Pmet* represents the metallostatic pressure. The shrinkage pressure, *Pretrait*, is caused by the contraction of the metal during solidification. The surface pressure *Psurface* is defined as the interface pressure between the gas bubbles and the surrounding metal.

Zou et al. [14] have proposed a mechanism to describe the evolution of the hydrogen concentration in a eutectic alloy during the formation of gas pores. The steps composing the gas porosity formation mechanism are as follows: Step 1: the liquid with hydrogen is gradually enriched. The amount of porosity that forms during this step is minimal. There is a continual increase in the solid fraction. Step 2: the hydrogen concentration in the liquid reaches a maximum value, which depends on the initial hydrogen concentration and the cooling rate of the metal. The porosities form between the secondary dendrite arms. Step 3: the hydrogen concentration decreases rapidly in the liquid due to the rapid development of hydrogen bubbles responsible for the formation of porosity. The pores grow rapidly, up to a maximum diameter. The solid fraction increases slightly. A 1% increase in the solid fraction contributes to approximately a 15% increase in porosity. Step 4: the hydrogen content in the liquid slowly decreases to a minimum value. The rate of porosity formation decreases due to the decreasing hydrogen concentration. The porosity variation with the solid fraction is almost linear. Step 5: The hydrogen concentration is low, but it is constant. The formation of porosity occurs as long as the part is not completely solid due to solidification shrinkage. The solid fraction increases, while the porosity is constant.

### 1.1. Solubility of Hydrogen in Aluminum

**Figure 2:**
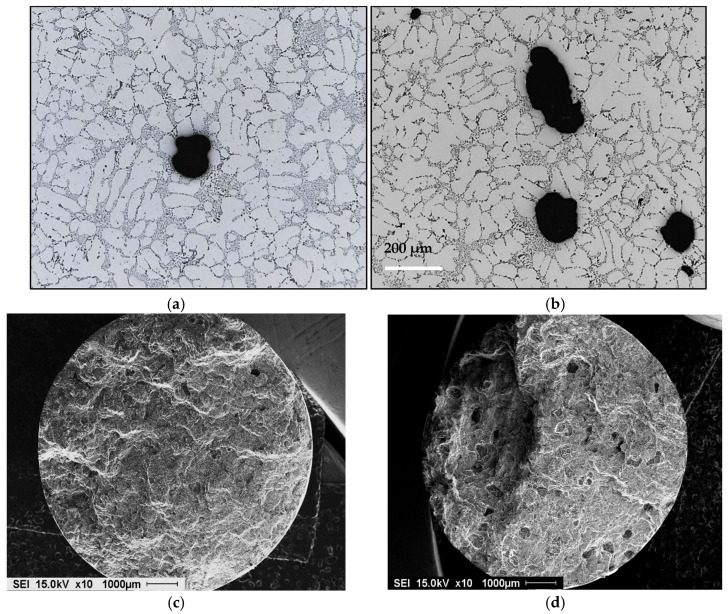
Optical microstructure of porosity in 319 alloy (**a**) low hydrogen 0.12 mL/100 g Al, (**b**) high hydrogen 0.25 mL/100 g Al, (**c**) and (**d**) fractured surfaces of tensile bars corresponding to (**a**) and (**b**), respectively.

The solubility of hydrogen in aluminum alloys is expressed as the amount of hydrogen that can dissolve in the metal bath. Solubility is expressed in milliliters of hydrogen per 100 g of metal and is influenced by three important factors: the temperature of the metal, the surrounding atmospheric conditions, and the chemical composition of the alloy [15,16], as shown in Figure 2.

According to Sieverts’ law, the solubility of diatomic gases (such as H_2_, N_2_, and O_2_) in metals is proportional to the square root of the partial pressure of the gas in thermodynamic equilibrium [17]. The internal gas pressure is determined using the concentration of hydrogen and Sievert’s law. The pore radius is governed by the interdendritic space (*r* = DAS/4), and the condition of porosity formation can be expressed according to the following relationship:

(2) $$Pg=Pint=Pext=Patm+Pmet+Psurface\;\left(=\frac{2\sigma }{r}\right)+Pretrait$$

Porosity pressure, N/m^2^,

Pg: Pressure inside the porosity, N/m^2^,

Pint: Pressure outside the porosity, N/m^2^,

Patm Atmospheric pressure, N/m^2^,

Pmet: Metallostatic pressure, N/m^2^,

Psurface: Pressure induced by surface tension, N/m^2^,

Pretrait: Pressure induced by metal shrinkage, N/m^2^.

**Figure 3:**
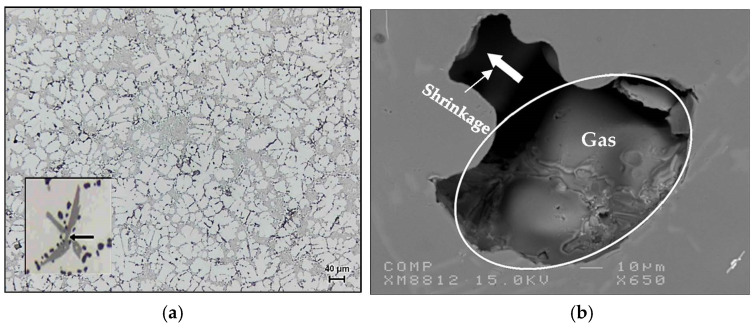
(**a**) Optical micrograph of 354 alloy containing 0.4% Zr - inset is an (Al,Si)_3_(Zr,Ti) phase particle (500×), arrowed; (**b**) backscattered electron micrograph of 413 alloy showing the branching of a gas pore into a shrinkage pore during the solidification process.

The solubility of H_2_ in aluminum-based alloys depends as well on the chemical composition of the alloy. Anyalebechi [5] studied the hydrogen solubility in Al-H-X alloys where X = Cu, Zn, Fe, Mg, Ti, or Li. The results show that plots of log_10_ H vs. wt.%X revealed that isothermal hydrogen solubility in liquid Al-H-X alloys at 101.3 kPa hydrogen partial pressure decreases with increase in Cu, Si, Zn, and Fe levels but increases with increasing levels in Mg, Li, and Ti. Another study by Safyari et al. [18] showed that coherent Al_3_Zr dispersoids in the matrix lead to superior hydrogen embrittlement resistance of the alloy. Figure 3 shows examples of porosity in two alloys with different Si content.

**Figure 4:**
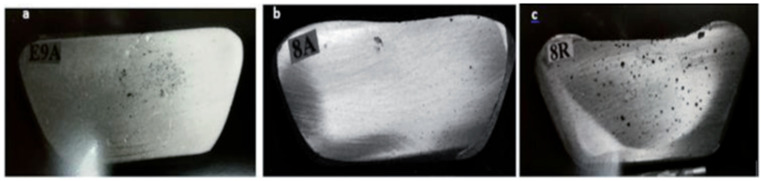
RPT test taken from melts of (**a**) as-received 319 alloy; (**b**) after degassing; (**c**) melt containing 0.25 mL/100 g Al.

As Sr has a high affinity to react with oxygen to form SrO oxides, Sr was added during the last 10 min of degassing. As for varying the hydrogen level, small pieces of raw potato were added to the melt at the end of degassing (in which Sr was not used), followed by an AlScan™ measurement of the hydrogen level attained in the melt [19]. In addition, reduced pressure test (RPT) samplings were taken from the melts. Figure 4 shows examples of RPT samples sectioned in half to examine the porosity observed under different melt conditions. In all cases, samples for chemical analysis were also obtained from each melt prior to pouring.

**Figure 5:**
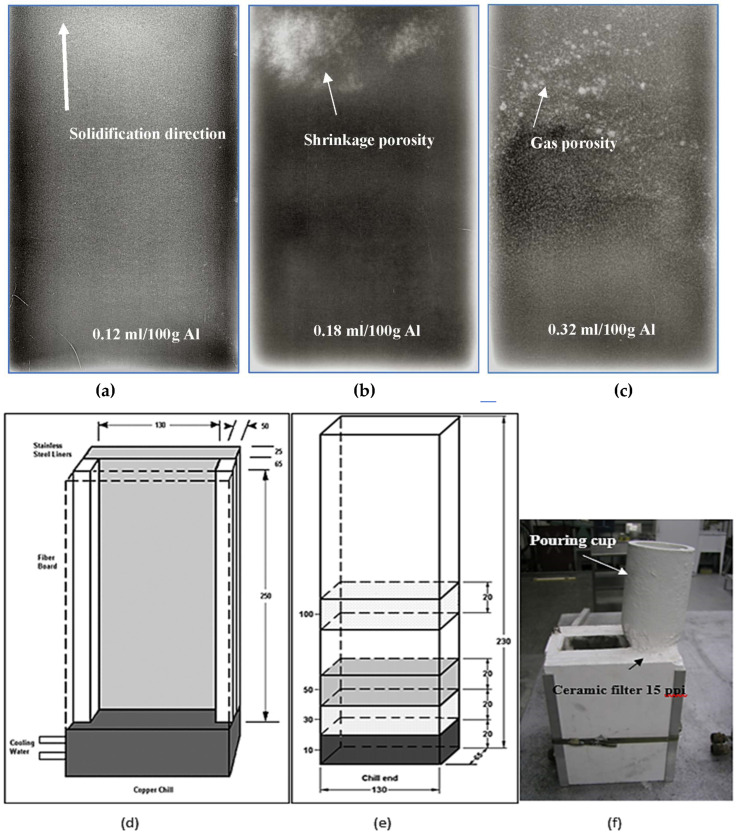
(**a**–**c**) Radiographic images of porosity distribution at different H_2_ levels: (**a**) 0.12, (**b**) 0.18, (**c**) 0.22 mL/100 g Al, (**d**,**e**) schematic diagram (all dimensions are in mm), (**e**) actual mold configuration, and (**f**) typical casting.

**Figure 6:**
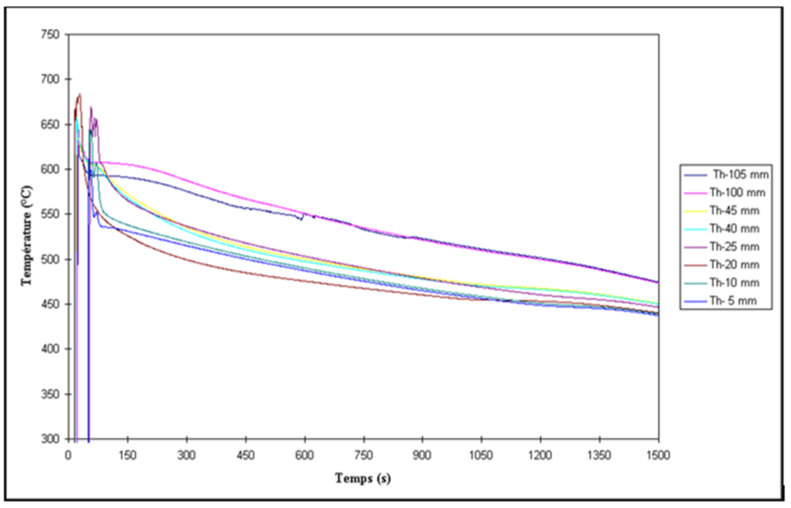
Temperature–time curves obtained at different distance from the chill end. Thermocouples were placed along the centerline of the mold through the refractory block into the cavity.

**Figure 7:**
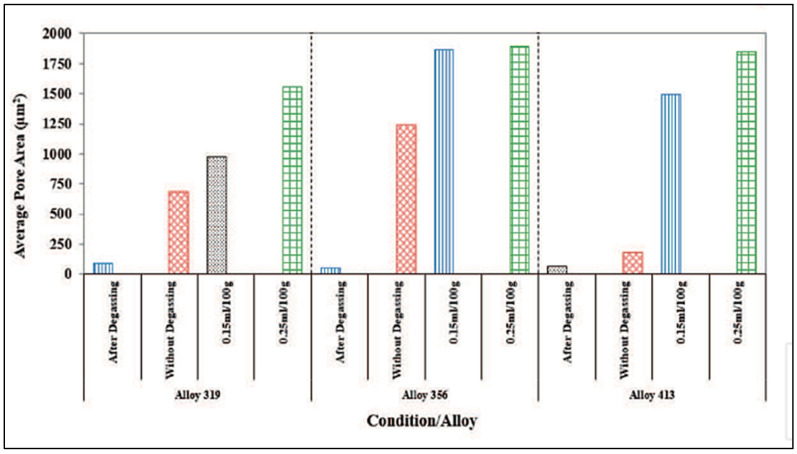
Comparison of melt treatment and hydrogen level on the pore size obtained in three Al-Si based alloys, highlighting the effect of oxides as a function of the total amount of added Sr and grain refiner and hydrogen level.

Abdelaziz et al. [20] investigated the effect of H_2_ content coupled with solidification rate, using a directional solidification technique. Figure 5 shows different radiographs taken of castings obtained using this technique, using melts containing various hydrogen levels. The light areas in Figure 5a represent zones of low H_2_ absorption, which are generally non-porous areas. The observed porosity in Figure 5b is caused by metal shrinkage during solidification. The solidification front is directed upwards and concentrates the shrinkage at the top, where the metal is the last to solidify. Metallographic analysis showed that the proportion of porosity varied with the solidification rate, i.e., with the distance away from the chill end (bottom) of the mold. The X-ray radiograph of the casting shown in Figure 5c highlights this phenomenon. The pore size and concentration in the bottom part of the casting are much lower than in the top. As the metal solidifies, hydrogen loses its ability to dissolve in the solid metal and, when moving upwards, results in increasing the concentration and the size of the pores in the upper zones. Figure 5d–f show the details of the used mold, whereas Figure 6 depicts the temperature–time curves obtained along the side of the mold. Figure 7 summarizes the effect of melt treatment and hydrogen level on the pore size obtained in different Al-Si-based alloys [19].

### 1.2. Effect of Temperature

Temperature is the most important factor that influences the solubility of hydrogen. The dissolution of hydrogen is approximately twenty times higher in the liquid state than in the solid state. The discontinuity in the solubility of hydrogen is observed during the solidification of the metal. The hydrogen dissolved in the metal bath is suddenly rejected through the pasty zone during solidification, which allows the formation of porosities. The solubility of hydrogen after fusion continues to increase as a function of temperature. On the other hand, the work of Poirier et al. [21] demonstrates the use of a linear-regression technique to determine the solubility of hydrogen in aluminum–copper alloys. From Hoff’s equation and Poirier’s regression equation, the following equation can be obtained:

(3) $$\log S=-\left(\frac{A}{T}\right)+B$$

where *S* is the solubility, in mL of hydrogen per 100 g of metal at a pressure of 1 atm of hydrogen gas; *A* and *B* are parameters dependent on the copper concentration; *T* is the temperature in K.

### 1.3. Effect of Atmospheric Pressure

The hydrogen contained in the ambient air exists in the H_2_ form. When it is dissolved in the metal, the hydrogen is transformed into the 2H form. At constant temperature, the solubility of hydrogen depends on the partial pressure of this element with the metal bath. [22] established an equation based on Sievert’s’ law that expresses the solubility of hydrogen as a function of the partial pressure of hydrogen in the atmosphere and the atmospheric pressure:

(4) $$S=625\;\sqrt{Ph2/Pa}\times 10^{-2760/T}$$

### 1.4. Effect of the Chemical Composition of the Alloy

**Figure 8:**
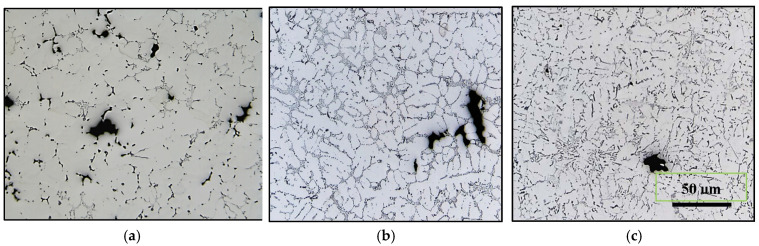
Variation in shrinkage porosity as a function of Si content: (**a**) 0.5% (220 alloy), (**b**) 8.5% (380 alloy), and (**c**) 11.5% (413 alloy).

The chemical elements used in aluminum alloy have an influence on the solubility of hydrogen in aluminum. Among the common alloying elements used with aluminum (Al), silicon (Si), manganese (Mn), and nickel (Ni) decrease the solubility of hydrogen, iron (Fe) and chromium (Cr) have no significant effect, while magnesium (Mg), titanium (Ti), and zirconium (Zr) increase the solubility of hydrogen. On the other hand, the investigations of Dong et al. [23] reveal that the shrinkage porosity is influenced by the percentage of Si present in the alloy used. Figure 8 shows the variation of the shrinkage porosity according to the quantity of Si present in the alloy. According to [24] with the increase in Si, the decrease in porosity formation that results from the decreasing solidification interval and increasing fluidity of the alloy due to the increase in Si content is superior to the increase in porosity formation by a slight coarsening of the grain size. Hydrogen is absorbed in liquid aluminum according to the following reaction:

(5) 2Al(l)+3H2O(g)=Al2O3(s)+6H1

### 1.5. Pore Nucleation 

When the concentration of hydrogen in the interdendritic liquid is at a sufficient level to create a hydrogen pressure exceeding the sum of the pressure inside the interdendritic liquid, the pressure induced by the surface tension, and the shrinkage pressure, the hydrogen bubble gives way to the porosity that can form during the solidification of the metal. In general, the concentration of hydrogen in the liquid is inversely proportional to the fraction of the liquid, due to the phenomenon of diffusion of hydrogen in the liquid metal, which occurs very rapidly. There is then an increase in the concentration of hydrogen, or even at the limit, an overrun of the solubility limit. The enrichment continues until the gas pressure is higher than the local pressure, which allows the nucleation of the hydrogen bubble. When the maximum is reached, at this precise moment, the germination of the porosity begins. 

Two different nucleation mechanisms then occur: heterogeneous nucleation and homogeneous nucleation. Due to the surface tension, the homogeneous nucleation mechanism is very difficult and requires very high excess pressure. Due to this difficulty, the nucleation of porosities takes place almost exclusively on heterogeneous nucleation sites. Heterogeneous nucleation is mainly due to the contact of the metal with the walls of the mold, inclusions, and the presence of gas bubbles. The porosity formation criterion can be defined as a pressure balance according to the following conditions [21,22,23,24,25]:

(6) Pg=Pext=Patm+Pρ+Pσ+Ps

where *Pg*: Porosity pressure, N/m^2^,

*Pext*: Pressure outside the porosity, N/m^2^,

*Patm: Atmospheric pressure, N/m2,*

*Pρ: Metallostatic pressure, N/m2,*

*Pσ: Pressure induced by surface tension, N/m2,*

*Ps: Pressure induced by metal shrinkage, N/m2,*

**Figure 9:**
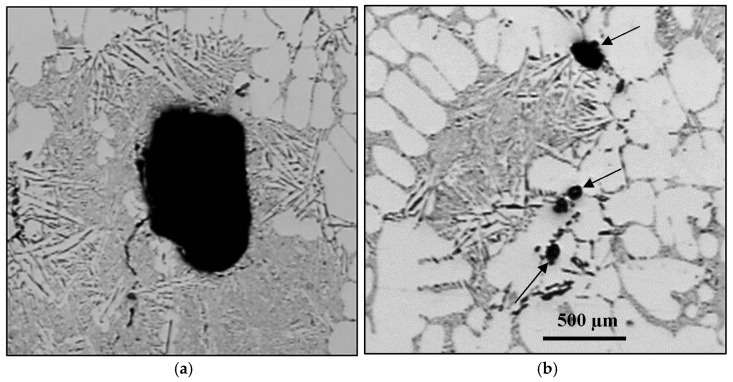
(**a**) Precipitation of coarse porosity in the interdendritic region and (**b**) microporosity at edges of the Al grains (arrowed black).

Nucleation of porosity can be possible when the pressure inside the liquid zone becomes equal to the outside pressure. In addition, with moderate gas pressure, porosity may be formed if the radius of curvature is high or the surface energy is low. The porosity forms at the base of the secondary dendrite arms, which correspond to the position of the heterogeneous sites. Additionally, inclusions can also reduce the surface energy. This indicates that if the fluidity is good, the interdendritic liquid supply will be improved, the pressure drop caused by the microshrinkage will be reduced, and the formation of pores will thus be limited, as shown in Figure 9.

## 2. Elements Influencing Porosity Formation

### 2.1. Elements Producing Modification and Refining

To modify the grain structure and improve the characteristics of the alloy, certain elements may be added to the base alloy. The use of a titanium–boron (Al-Ti-B) master alloy makes it possible to produce grain refinement. The addition of Sr allows the modification of the eutectic Si flakes into a more fibrous structure.

### 2.2. Grain Refining (Ti-B)

**Figure 10:**
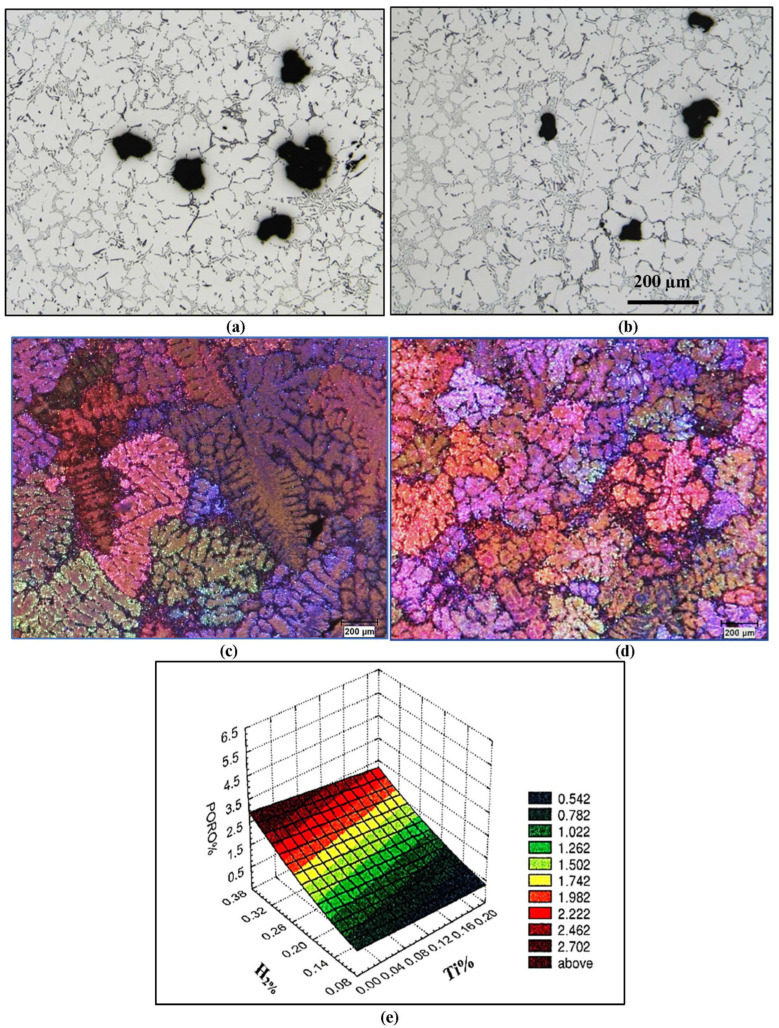
Optical microstructure of 319 alloy: (**a**) before grain refining; (**b**) after grain refining (using 0.0.15%Ti in the form of Al -5%Ti-1%B); (**c**) grain size in (**a**); (**d**) grain size in (**b**); (**e**) Porosity-H_2_-Ti relationship.

Grain refining is a process by which an Al-Ti-B master alloy is added to the molten metal bath to obtain a more refined Al-grain structure. This transformation mechanism aims to disperse the porosity more finely, by standardizing the mechanical properties throughout the casting. The use of grain refining demonstrates that the grain size has a significant influence on the pore size, as most pores are located around the edges of the grains. Thus, the use of grain refinement, by decreasing the size of the grains, will have a direct effect on the size of the pores. Investigations carried out by Tylenius et al. [26] report the following remarks: “The refining of the grain decreases the maximum length of the pores while the surface porosity and the density of the pores increase, the maximum area of the pores remains unchanged.” The use of Ti-B for grain refining Al-6%Si-2%Cu alloy allowed Mohamed et.al. [25] to obtain a reduction in porosity of the order of 0.7% to 2%. Figure 10 demonstrates examples of how (a,b) the porosity is reduced when (c,d) grain refining 319 alloy as obtained from our studies [20]. Figure 10e shows the inter-relationship between percentage porosity, hydrogen content, and grain refining (in terms of Ti content) obtained from an extensive study carried out by our group [1].

### 2.3. Modification with Strontium Addition

The use of strontium (Sr) makes it possible to modify silicon-based aluminum (Al-Si) alloys. The modification of Al–Si alloys with Sr is a process used in foundries, which makes it possible to change the eutectic silicon from a coarse plate-like to a more fibrous and refined form. The advantages related to Sr modification are to increase the mechanical properties of the castings and to reduce the solution treatment time for possible heat treatment [1,2,26,27,28,29]. The conclusions from the various studies in the field of modification diverge greatly from each other. In general, the authors arrive at the same consensus, i.e., the use of Sr to modify the alloys increases and standardizes the porosity.

**Figure 11:**
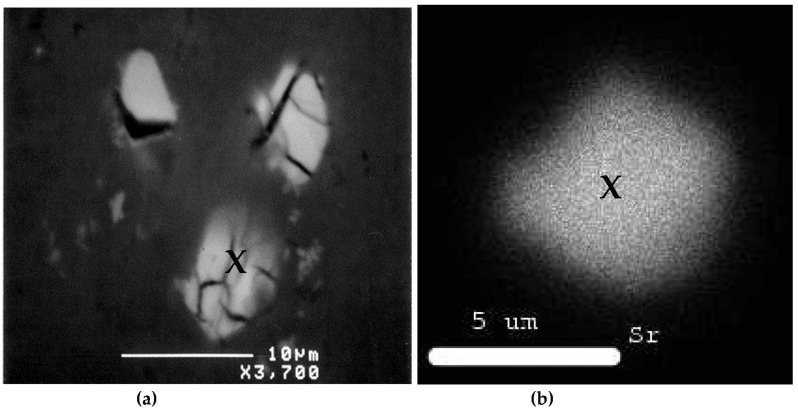
Example of porosity in Sr-treated alloy: (**a**) backscattered electron image showing Sr-rich particles within a pore; (**b**) X-ray of Sr distribution in the particle marked X in (**a**). Note the rounded shape of the Sr-rich particles in (**a**).

During solidification, in the interdendritic liquid of an unmodified alloy, there is a local pressure drop caused by the solidification contraction (shrinkage) and by a lack of supply of the liquid. This pressure drop offers the possibility of nucleation of a pore and facilitates the rejection of hydrogen in the interdendritic liquid. However, Miresmaeili et al. [30] reported that the modified alloys have a 4–10°C lower eutectic temperature, longer modification time, increased solidification time, increased dendrite volume fraction, an extension of the pasty zone, and reduction of the volume fraction of the eutectic liquid. These factors would result in an increase in the formation of microporosity. Examples of microporosity observed in Sr-treated A319.2 alloys [28] are shown in Figure 11.

Several authors [7,31,32,33,34,35] used an A356 alloy with a 0.03% Sr modification and hydrogen level constant at 0.13 mL H_2_/100 g Al, using a gas recirculation technique. They observed that the modification of Al alloy A356 promotes the formation of microporosity. Lee et al. [36], from their studies on Al-7Si-0.3%Mg aluminum plates, showed a correlation between the liquid flow in the interdendritic spaces and the liquid fraction. The results predicted an increase in the cooling range caused by the modification of the alloy, which had some influence on the formation of microporosities. Increasing the cooling range has the effect of widening the pasty zone, increasing the shrinkage pressure drop by 56%, reducing the liquid fraction from 0.52 to 0.46, and decreasing the interdendritic liquid velocity by 56%.

### 2.4. Thermal Parameters

In all the parameters used to evaluate porosity, we identified, among other factors, the thermal parameters related to the cooling condition of the metal. In addition to the temperature, these parameters are the speed of the solidification front, the solidification time, and the thermal gradient G.

#### 2.4.1. Solidification Rate

The solidification rate can be defined as the ratio of the distance between two points of metal parallel to the direction of the solidification front and the time the solidification front takes to travel this distance. The solidification rate can be considered a measure of the rate of solidification. According to Ohnaki et al. [36] and Sabu et al. [37], an increase in the solidification rate has the effect of decreasing the percentage of porosity even if the hydrogen concentration increases. This phenomenon is attributable to the fact that at a high solidification rate, the formation and growth of pores are limited by the small size of the dendrite arms, which renders pore nucleation more difficult. On the other hand, the work of Tylenius et al. [24] reports that the surface percentage porosity, the maximum surface or pore area, the density of the pores, and the maximum pore length increase with the increase in the speed of solidification.

#### 2.4.2. Solidification Time

**Figure 12:**
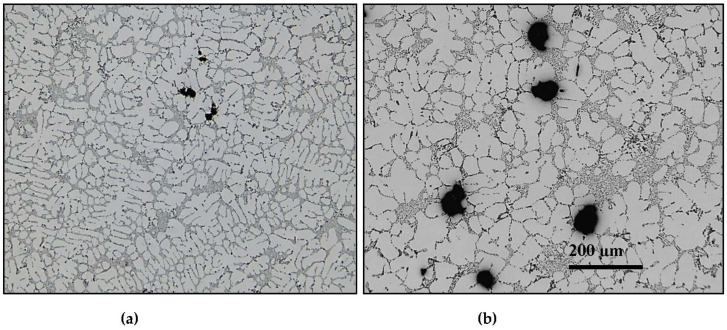
Optical micrographs of 319 alloy obtained at (**a**) solidification rate ~8 °C/s and (**b**) solidification rate ~0.35 °C/s.

The solidification time is defined as the time it takes for the metal to travel from the liquid to the solid state at a temperature of 500 °C. The use of a chilled base has the effect of promoting directional solidification and solidification time. The studies conducted by Khalajzadeh and Beckermann [38] and Ye [4] on thermal conditions during solidification establish a linear relationship between solidification time and distance from the base for castings carried out using a directional solidification mold (Figure 5). Moreover, the results obtained from these investigations make it possible to observe an increase in the porosity when the solidification time increases while moving away from the base of the mold. Depending on the position in the mold from the base (or solidification time), the authors established a relationship that characterizes the amount of porosity:

(7) $$p=a+k.d^{n}$$

where *p* is the porosity value, *a* is a constant associated with the solidification rate (0: directional solidification mold; 1: sand mold), *k* is the slope of the metal cooling curve, which increases for a slow solidification time, *d* is the distance which separates the sample from the base of the mold, and *n* is equal to 1 for very short solidification times and >1 for a long solidification time, as demonstrated in the optical micrographs shown in Figure 12.

#### 2.4.3. Thermal Gradient

The thermal gradient can be defined as being the ratio of the temperature difference between two fixed points over the distance that separates these two points. The work of Pan et al. [39] has made it possible to establish a relationship that links the thermal gradient G and the porosity volume fraction through the following relationship:

(8) $$p_{vol}=1.308-0.633\times \log\left(G\right)$$

## 3. Techniques Used for Porosity Characterization

There are three main categories of techniques or approaches used for the evaluation of porosity characterization. The first category encompasses studies that use an experimental approach to evaluate porosity, based on specific characteristics of pore development. The second category includes research that deals with porosity using mathematical models. The third category entails the quantitative prediction of porosity using statistical reduction techniques.

### 3.1. Evaluation of Porosity Using Experimental Measurements

The experimental evaluation technique is an approach based on the use of certain parameters to describe the quantity and the number of pores, namely, the combination of certain criteria and the use of different thermal parameters such as solidification time, thermal gradient, and solidification rate. The work of a number of scientists [40,41,42,43,44] on rectangular plates of an Al-7Si-0.3 Mg alloy with a reservoir size variation of 4 to 11 cm. The hydrogen content was kept below 0.01 mL/100 g, and the alloy was refined using an Al-5Ti-1B master alloy. Using a theoretical model for interdendritic filling and for the phenomenon of nucleation and porosity growth, they report that from the different observations, the solidification time alone cannot be considered an independent variable to characterize the interdendritic filling. The variation of the geometry of the reservoir has a great influence on the thermal variables, which govern the filling of the mold and on the distribution of the porosity. 

On the other hand, the work of Gu et al. [45] on Al–Si alloys, with a Si content varying from 4 to 8%, degassing of the alloy by vacuum, a Sr modification of 500 ppm, and a sodium (Na) modification of 0.2%, found that an increase in the Si content limits filling and increases shrinkage defects during solidification. In addition, the use of Na substantially reduces the porosity. Jang et al. [46] focused their work on Al-Si alloys with Si contents ranging from 1.7 to 12% Si and the amount of hydrogen varying from 0.2 to 0.8 mL/100 g. of metal. The experiments were carried out using cylindrical sand molds to evaluate the density of the castings. The percentage of porosity in the parts was calculated on the basis of the theoretical densities considering the constituents of the alloy and using the rule of mixtures. 

The first assumption made in the analysis of the results was that the density of the samples contains almost all of the gases present in the metal. The second assumption concerned the use of the type of mold, which justifies that the maximum density of the parts is caused by the release of gases and not by the shrinkage of metal. Finally, the third hypothesis, by far the most important, concerned the fact that, when casting is carried out at a slow solidification rate, the gas that was originally dissolved in the metal separates and forms the porosity, and that the majority of the gas is found in the pore except for the fraction, which remains soluble in the metal.

Argo and Gruzleski [47] investigated porosity in modified Al alloys on Tatur specimens using A356.2 alloy with a constant hydrogen level at 0.2 mL/100 g of metal. The modification of the alloy was achieved by adding Sr varying from 0 to 360 ppm. Radiographic analysis of the Tatur specimens revealed that the modified samples tended to develop higher shrinkage microporosity, compared to the unmodified samples. Moreover, from these observations, they noticed that the modified specimens have less tendency to develop macroporosity. The results obtained were verified using density measurement of the Tatur test bars. In contrast, results obtained by Brůna and Sládek [48] using the Tatur mold revealed the presence of severe shrinkage porosity in their 356 alloy casting, since the mold has no external riser.

### 3.2. Evaluation of Porosity Using Mathematical Modeling

The evaluation of the porosity by the use of mathematical modeling makes it possible to understand all the implications underlying the mechanisms that control the formation and growth of the porosity. To obtain rigorous experimental validation, a limitation of the solidification parameters must be considered. The specific use of a type of permanent mold or a sand mold is very important for the control of the solidification parameters. When using a mathematical model, several unknown variables that govern the nucleation, pore growth equations, and the permeability of the interdendritic network must be considered constant.

Significant work has been conducted by Zou et al. [14] on the modeling of microstructure evolution and porosity formation. The modeling was carried out using A356.2 alloy, and the hydrogen concentration was kept constant at 0.65 mL/100 g Al. In the modeling/evaluation of volume percent porosity and pore size, several parameters were affected. The permeability (*K*) of the dendritic network (*DAS*) was obtained using the following equation:

(9) $$K=\frac{D_{g}\;.\;DAS_{2}^{1/3}.d_{g}^{2/3}.{\left(1-f_{s}\right)}^{2.237}\;}{2*6.35.10^{-3}{\left(fs\right)}^{0.926}}$$

where *f_s_* represents the fraction of liquid and *D_g_* is the gas pore diameter 

The results obtained from the modeling did not consider the modification and the refining of the Al grains, parameters such as the size of the grains, the interdendritic spacing (DAS), and the area of the Al-Si eutectic. The number and dimension of the pores are comparable with other similar experimental results [10]. The main characteristics observed from the modeling are the following: (1) the formation of pores does not occur below a critical hydrogen concentration threshold; (2) the initiation of the formation of porosity takes place in the first phase of eutectic solidification and continues till the fraction of the solid phase at 43%; (3) the number of pores in the metal is inversely proportional to the cooling rate and directly proportional to the initial hydrogen content. Moreover, if the cooling rate is greater than 5 °C/s, the number of pores formed is mainly controlled by the hydrogen concentration. 

The mathematical model described above highlights the fact that the factor that mostly influences the porosity, is the hydrogen content present in the metal. The use of mathematical modeling has enabled Kubo and Pehlke [49], Carlson et al. [50], and Kuznetsov and Vafai [51] to develop a tool to evaluate the size of the pores and the volume percentage of porosity. The model was developed by comparing experimental data obtained from molded plates of Al-4.5% Cu aluminum alloy. The various parameters used for the modeling are given in the following equation used for evaluating the permeability of the alloy:

(10) $$K=\frac{{\left(DAS\right)}^{2}\;.\;{\left(f_{L}\right)}^{3}\;}{180\;{\left(1-f_{l}\right)}^{2}}$$

where $f_{l}$ represents the fraction of liquid and *DAS*, the dendrite arm spacing.

Hydrogen solubility varies with hydrogen pressure, and Sievert’s law, gas pressure, *Pgas*, is assessed using the liquid pressure (*Pliq*) and the surface pressure (*Psurf*). The pore growth and nucleation conditions are estimated with the use of the ideal gas law, the law of conservation of mass with the following equation:

(11) $$P\geq \;P_{liq}+P_{surf}$$

The results obtained from this modeling work are representative of the experimental work. Modeling of the solidification mechanism has revealed that the pressure differential and the porosity number increase as the liquid fraction tends towards zero. In addition, the model demonstrates that hydrogen evolution and metal shrinkage are the key mechanisms responsible for porosity formation.

Anyalebechi [52], on the other hand, focused their modeling work on the volume percentage and the dimension of the pores observed in A356.2 alloy. In addition, grain refining and Sr modification treatments were previously carried out on the alloy, and the hydrogen concentration was kept constant at 0.58 mL/100 g of metal. To properly model the phenomenon, they considered unidirectional thermal conditions, where the pressure variation was evaluated according to the following formula:

(12) $$\Delta P=\frac{\beta }{1-\beta }.\;\mu .V.L.\;\frac{\ln\left(g_{e}\right)}{\gamma \left(1-g_{e}\right)}$$

where *β* is the volume shrinkage factor; *V* is the rate of solidification; *µ* is the viscosity; *L* is the length of the mushy zone; *g_e_* is the volume fraction of the eutectic after casting; *γ* is the permeability of the mushy zone.

They further considered that pore nucleation takes place when the pore radius is 1 µm and that pore growth is due to hydrogen rejection and solidification shrinkage, assuming that the gas pressure is greater than the sum of the liquid and surface pressures. 

**Figure 13:**
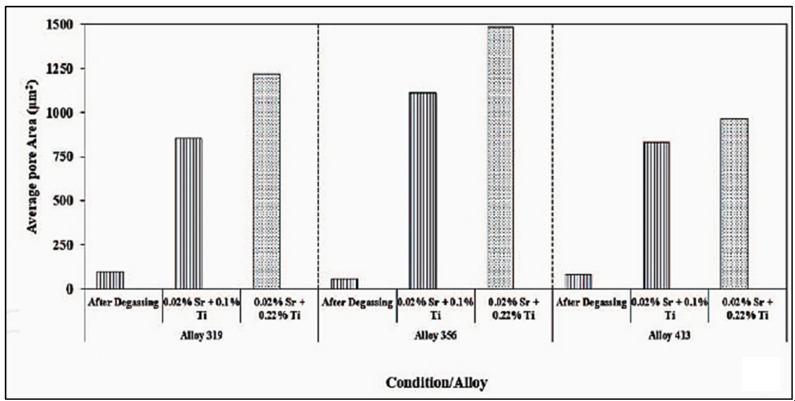
Effect of total amount of added Sr and grain refiner on the average pore size in three Al–Si based alloys.

**Figure 14:**
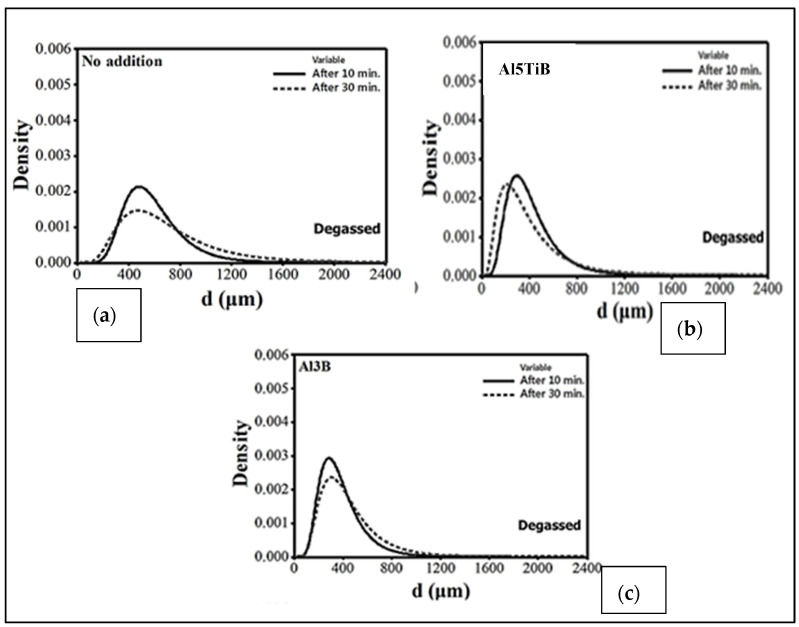
Lognormal distributions of pore sizes in Al-7%Si-0.35%Mg (A356) alloy after 10- and 30-min holds: (**a**) for non-treated alloy; (**b**) with Al-5Ti-1B addition; (**c**) with Al-3B addition. In all cases, the alloy melts were degassed to avoid entrapment of oxide films.

**Figure 15:**
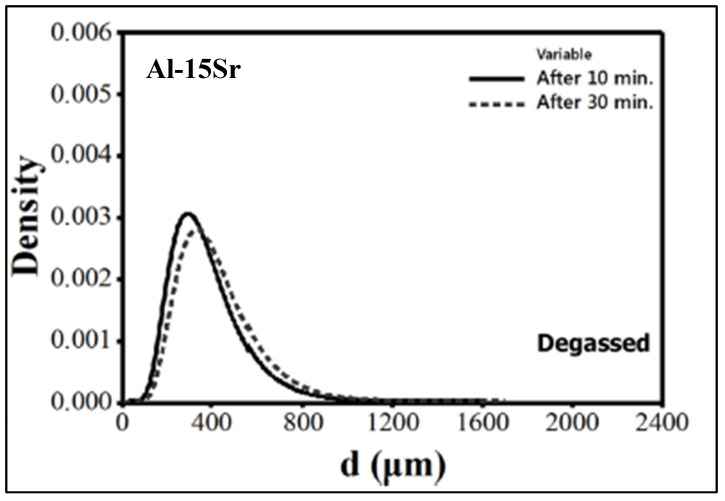
Lognormal distributions of pore sizes for Al-15Sr addition after 10- and 30-min holds.

Figure 13 exhibits the pore diameter as a function of the grain size for the experimental results revealing that the porosity of the A356.2 samples is inversely related to the grain size and decreases as grain size increases. The results obtained were used as input data for constructing the mathematical model. From the model obtained, the main conclusions noted were that (i) for a given level of hydrogen, the volume fraction and the pore size decrease with the increase in the solidification rate; (ii) for a constant solidification rate, the volume fraction and the pore size decrease with lowering of the hydrogen content. The use of grain refining allowed for a reduction in the porosity volume fraction and pore size and a more uniform distribution of porosity. In general, grain refiners have a different effect on the porosity formation of Al-Si alloys with regard to their solidification morphology [53]. On the other hand, the use of Sr for the modification significantly increased the volume fraction and the dimension of the pores as shown in Figure 13. Figure 14 displays lognormal distributions of pore sizes for Al5Ti1B and Al3B addition after 10- and 30-min holds [54]. As can be seen, porosity sizes are skewed towards smaller pore size (d), reaching a maximum at around 400 µm. Also, no differences are noted between the two types of grain refiners used. Figure 15 reveals a similar pattern with the addition of AlSr15 master alloy. It should be noted that holding time has a marginal effect on the pore size. The mean of a lognormal distribution is found by:

*d*_eq_ = e^µ + σ2/2^ (13)

where σ is the shape and µ is the scale parameter [55,56]. In general, µ is approximately 13 and σ is approximately 0.73.

### 3.3. Evaluation of Porosity Using Statistical Reduction Techniques

Evaluation by the use of a statistical reduction technique makes it possible to determine certain variables (responses) according to various control parameters (variables). The results that allow this technique of statistical reduction are an empirical quantitative model, which from experimental data, transmit information on the quantity, the dimensions, and the characteristics of the porosities. Jolly and Katgerman [57] conducted an experimental study concerning the use of the multiple-regression technique for the prediction of microporosity in castings of A356 alloy. More than 300 experiments were carried out, and several parameters were analyzed. The main parameters used were the thermal gradient at 10 °K below the solidification point of the metal (solidus), the solidification rate at the liquid-solid interface, the solidification rate at 10 °K below the solidification temperature, and the level of hydrogen present in the metal. 

The evaluation of the porosity characteristics was carried out using an image analyzer, where parameters such as pore density, maximum pore diameter, and pore size distribution were determined. From the results, a very good correlation was obtained between the thermal gradient parameters and the hydrogen content to define the resulting porosity, where 75% of the variation in porosity was explained by the effect of the three parameters, i.e., the thermal gradient at the end of solidification, the rate of solidification, and the hydrogen content. The density of pores could be expressed linearly by these three parameters. As shown in Figure 5, one can clearly notice the effects of hydrogen content and rate of solidification on the percentage of porosity.

Tynelius et al. [26] also used the multiple regression approach to analyze their results. The major advantage of this statistical technique stems from the fact that one can obtain a response parameter from several complex prediction variables. In addition, this technique allows the use of different molds, regardless of their geometry. Consequently, this advantage makes it possible to reproduce as efficiently as possible the effect of the various variables on the porosity characteristics. 

The various statistical models presented in the literature are all based on data accumulated through experiments. From the different results obtained, some models have been developed. Table 1 presents the evolution of the model used to predict the maximum pore size. Thus, the final model obtained explains 73% of the total variance in the data collected. The variables included in the model are the interactions between the hydrogen content and the solidification time, the Sr concentration, the solidification rate, and the amount of grain refiner used. The final model takes the form of the following equation:

Maximum pore size (μm) = A([H] × [t_s_]) + B([H] × [Sr]) + C([H] × [V_s_]) + D([H] × [Ag]) (14)

where A, B, C, D are model coefficients; 

[H] × [ts] is the interaction between hydrogen content and solidification time;

[H] × [Sr] is the interaction between hydrogen content and strontium concentration;

[H] × [Vs] is the interaction between hydrogen content and solidification rate; and

[H] × [Ag] is the interaction between hydrogen content and grain refiner.

From the model, certain iso-contour curves were obtained to represent the effect of the predictor variables on the observed parameters. Many models have been developed using the multiple-regression technique, based on observed experimental data, from which several observations could be noted. Among the thermal parameters, the solidification time and rate are found to be the most representative variables. 

**Figure 16:**
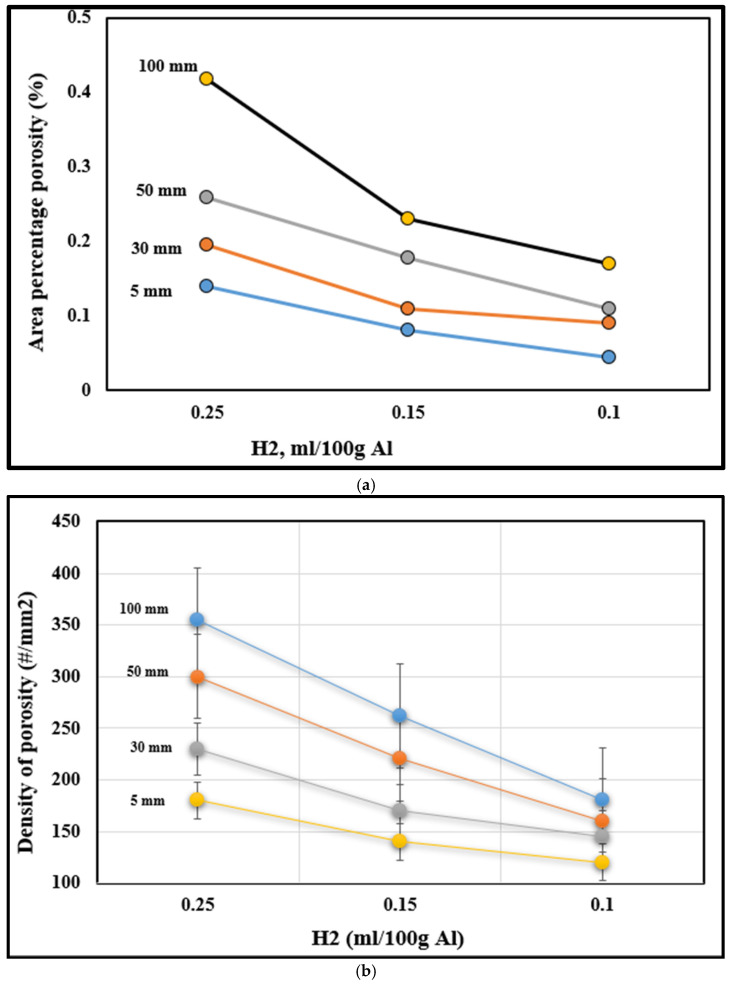
Variation in (**a**) porosity percentage and (**b**) density of porosity as a function of distance from the chill end.

The hydrogen concentration (Figure 16) is considered the most influential predictor parameter among all the other parameters. Moreover, the interaction of hydrogen with the other predictive variables forms sets that correlate very strongly in the model, and this for the majority of the observed variables. Similarly, Sr is a predictor variable that strongly affects models of percent porosity, maximum pore size, and maximum pore area. To obtain a generalized model for different molds, particular attention must be paid to clearly differentiate the effects of the geometry of the mold from the thermal effects imposed by the geometry of the mold. Finally, the multiple-regression technique does not make it possible to establish a model for all the variables that one wishes to deal with at the outset [58].

## 4. Analysis of Statistical Data

To obtain distinctive and comparative results for end chill mold, we applied the multiple-regression technique independently of the type of mold. The multiple-regression technique is applied to 135 cases for the directional solidification mold. The multiple-regression technique consists of a series of independent variables, which can be written in the form:

$$y_{i}=\beta_{0}+\beta_{1}x_{1i}+\beta_{2}x_{2i}+\ldots +\beta_{p}x_{pi}+u_{i}\;.\;i=1,\;2,\;\ldots ,\;n$$

where *β_0_*, *β_1_*, …, *β_p_* are the regression coefficients, and *x_1_*, *x_2_*, …, *x_p_* are the independent variables of the model.

The software used to process all the data is the multiple-regression sub-software of the statistical software Statistica™. The main parameters considered are the coefficient of determination *R^2^*, the overall test *F*, the marginal contribution t for each variable in the model, and the statistical significance variable *p* (*p*-level). The coefficient of determination R^2^ is written in the following form:

(15) $$R^{2}=\frac{\sum^{\;}{\left({\hat{y}}_{i}-\bar{y}\right)}^{2}}{\sum^{\;}{\left(y_{i}-\bar{y}\right)}^{2}\;}$$

The coefficient of determination is easily interpreted as being the proportion of variability explained by the regression equation [57]. If the coefficient of determination is close to 1, the fit is considered good. On the other hand, if the adjustment approaches 0, it means that the terms of the regression equation are not acceptable. The global test *F* is used to determine if there is a linear relationship between y and x. To this end, we must test the significance of the coefficient of determination. The overall test is to calculate the quantity:

(16) $$F=SR/p/s^{2}$$

where *SR* or the Sum due to regression = (*ST-SCE*), and it is compared to the following:

(17) F(p,n−p−1;α); 

The explanation of the variables is presented in the following analysis of variance Table 2.

By applying the result of *F*, it is possible to establish whether the value of the coefficient of determination *R^2^* is valid. We then associate low values of *F* with values of *R^2^* close to 0 and high values of *F* with values of *R^2^* close to 1 [58]. 

The marginal contribution *t* is a variable that follows a Student T-distribution and that expresses the difference between the errors for the model including the variable and the errors for the model that does not contain the variable. To justify the involvement of a variable in a model, the value of *t* will be high for a good justification and will be low for a non-justifying variable with respect to the model. The statistical significance variable *p* is considered a measure of the reliability of a variable with respect to the model. A high value of the variable *p* means that the variable is not significant in relation to the model. On the other hand, a very low value of the variable *p* indicates a very good reliability of the variable with respect to the model. In the various fields of statistical research, the acceptable limit value of *p* is of the order of 0.05.

The statistical results obtained from the directional solidification mold shown in Figure 5 allow us to describe different models for the majority of the parameters that describe the porosity. A statistical model equation was obtained for the following parameters: percent porosity, maximum pore area, average pore area, maximum pore length, average pore length for the irregular part of the distribution of the large pores, and the density of the length of the exponential part, where the terms ‘exponential’ and ‘irregular’ refer to the two parts of the porosity distribution curve obtained from pore area pore length parameter measurements (using optical microscopy in conjunction with image analysis). The other dependent variables that describe the porosity were not considered because no representative model could be proposed from the results obtained. Table 3 presents the results of the regression coefficient R^2^, the marginal contribution *t*, the statistical significance variable *p*, and the model constant *cte*, obtained for each of the variables of the different models obtained for the directional solidification mold.

Considering the results obtained for the different models, and that a regression equation is considered valid if the multiple-regression coefficient R^2^ is greater than 0.70, the value of the marginal contribution *t* is high and the significance variable *p* is less than 5%, the following representative models are obtained: (i) the percentage of surface porosity that has a regression coefficient of 0.8383; (ii) the maximum area of the pores of the irregular part with a regression coefficient of 0.8754; (iii) the average area of the pores of the irregular part having a regression coefficient of 0.8374; (iv) and finally the average and maximum length of the pores of the irregular part with regression coefficients that are 0.8169 and 0.7745, respectively. Given the low values of the significance variable and the marginal contribution *t*, all of these models are considered acceptable. 

The first parameter for which a linear regression model was established was the surface porosity percentage. The model can be written as a linear equation containing the following variables:

(18) $$Percentage\;surface\;porosity\;\begin{array}{l} =(-1.70931+3.0905\;\left[H\right]+0.01042\;\left[DAS\right]-0.00144\;\left[Sr\right]-1.0823\;\left[Ti\right]+6.8562\;\left[Mg\right]\\ +0.37737\;\left[Cu\right]-0.00026\;{\left[Ts\right])}^{2} \end{array}$$

where [H] represents the hydrogen content,

[DAS] is the value of the dendrite arm spacing, 

[Sr] is the strontium content,

[Ti] is the titanium concentration,

[Mg] is the concentration of magnesium,

[Cu] is the copper concentration, and

[Ts] is the solidification time.

**Figure 17:**
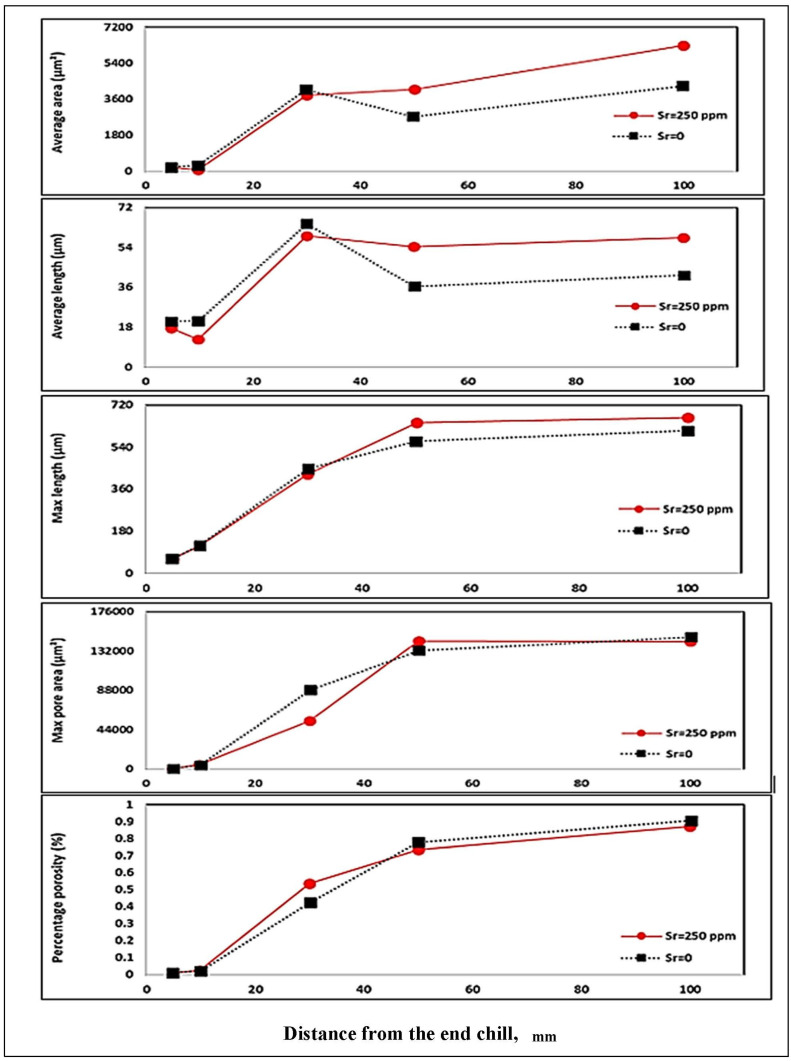
Porosity distribution in 319 alloy containing (H = 0.1 mL/100 g Al, Sr = 250 ppm, Ti = 0.2%) as a function of distance from the chill end.

**Figure 18:**
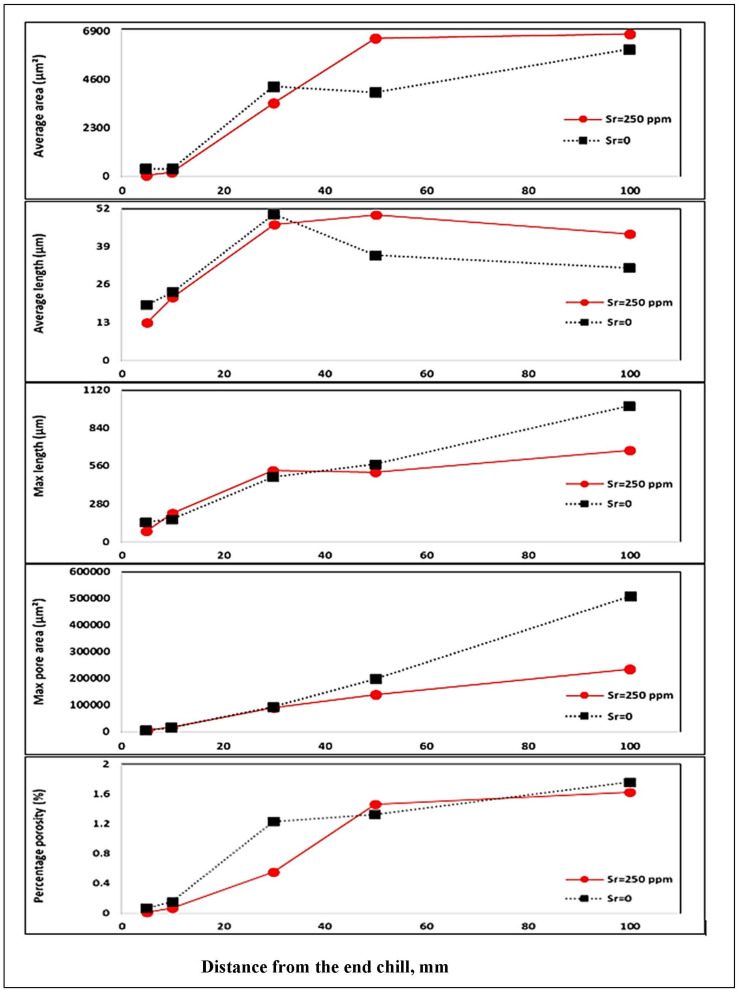
Porosity distribution in 319 alloy containing (H = 0.24 mL/100 g Al, Sr = 250 ppm, Ti = 0.2%) as a function of distance from the chill end.

The variable that mostly affects surface porosity is hydrogen. Hydrogen has a high marginal contribution at 9.336642, and the significance variable *p* is zero. The two other important variables that govern the model and have a good statistical significance are the interdendritic space and the concentration of Sr present in the alloy. In the case of the other variables, we consider that their effects are less important but necessary to keep the good model that comes closest to the experimental data. Figure 17 and Figure 18 depict the results obtained from the 319.2 alloy (grain refined and Sr -modified) at two levels of H_2,_ i.e., 0.1 mL/100 g Al and 0.25 mL/100 g Al, respectively [59,60].

## 5. Conclusions

Based on the results of statistical regression, the following points may be highlighted. First, there is a good agreement between the predicted and experimental results. Second, at very low H_2_, commencement of solidification is associated with the formation of shrinkage cavities in sections directly above the chill end (similar to hot zones), turning into more rounded ones as the solidification front moves upward. Third, increasing the concentration of H_2_ leads to the formation of spherical gas pores at the commencement of solidification, with an increase in the pore size as the distance from the chill end increases. In other words, the shrinkage porosity is converted into gas porosity. And finally, the mathematical models show that H_2_ is the main driving element in porosity formation compared to other parameters such as solidification rate, grain refining, or Sr modification.

## 6. Recommendation

Taking into consideration the analysis presented in this review, it is recommended that (1) statistical processing of results obtained from experimental data using different types of molds as well as real industrial castings is necessary to obtain a satisfactory model, which characterizes the porosity variables according to the constituents of the alloys and the solidification parameters, (2) in-depth analysis of the results and interpretation and justification of the different models for the end chill mold are essential, and (3) studying the effect of these parameters on the alloy mechanical properties (tensile, fatigue, creep, etc.) is deemed to be necessary.

## Figures and Tables

**Table 1:** Prediction parameters for maximum pore size using the statistical reduction technique.

**Response—Maximum Pore Size—Mold r^2^ = 0.64**		
**Variable of Prediction**	**T Value**	**Significance**
Interaction: Hydrogen & Solidification time Interaction: Interaction: Hydrogen & Strontium Concentration Interaction: Hydrogen & Grain Refiner Type of mold	16.37 5.04 3.04 N/A	0.0001 0.0001 0.0027 0.0162
**Response—Maximum Pore Size—Mold and Thermal r^2^ = 0.67**		
**Variable of Prediction**	**T Value**	**Significance**
Interaction: Hydrogen & Time of solidification	16.27	0.0001
Interaction: Hydrogen & Concentration of strontium	4.74	0.0001
Interaction: Hydrogen & Rate of solidification	3.5	0.0006
Interaction: Hydrogen & Grain refining	3.05	0.0027
Type of mold	N/A	0.3997
**Response—Maximum Pore Size—Thermal r^2^ = 0.73**		
**Variable of Prediction**	**T Value**	**Significance**
Interaction: Hydrogen & Time of solidification	19.68	0.0001
Interaction: Hydrogen & Concentration of strontium	5.83	0.0001
Interaction: Hydrogen & rate of solidification	4.47	0.0001
Interaction: Hydrogen & grain refining	3.23	0.0015

**Table 2:** Analysis of variance.

Origin of the Variation	Sum of Squares	Degree of Liberty	Average Square
Regression	*SR*	*p*	*SR/p*
Residual	$SCE=\sum^{\;}{\left(y_{i}-\hat{y_{i}}\right)}^{2}$	*n-p*-1	$s^{2}=\frac{SCE}{n-p-1}$
Total	$ST=\sum^{\;}(y_{i}-\hat{y}{)}^{2}$	*n*-1	

**Table 3:** Independent variables used for the description of the different models for the directional solidification mold.

Model	Independent Variables of the Model								R^2^
Directional Solidification Mold									
Dependent Variables	V	H	DAS	Sr	Ti	Mg	Cu	Ts	
Percentage of surface porosity	*t*	9.33642	7.8825	6.4751	−2.9226	3.6700	3.1143	−2.0987	
	*p*	0.000000	0.000000	0.000000	0.00411	0.000357	0.002283	0.037836	0.8383
	cte	3.0905	0.01043	0.00144	−1.0823	6.8562	0.37737	−0.0002	
Maximum area of pores (µm^2^) in the irregular part	V	H	DAS	Sr	Ti	Mg			
	*t*	8.61231	22.0702	4.71412	−3.6760	8.37343			0.8754
	*p*	0.000000	0.00000	0.000006	0.000347	0.00000			
	cte	5.94	0.0375	0.00245	−3.13	31.314			
Average area of pores (μm^2^) in the irregular part	V	H	DAS	Sr	Ti	Mg	Cu	Ts	
	*t*	6.08216	11.03291	7.01649	−3.7757	5.28969	2.47600	−2.7715	0.8374
	*p*	0.000000	0.000000	0.000000	0.000245	0.00000	0.014613	0.006425	
	cte	4.973	0.0360	0.00384	−3.454	24.410	0.7411	−0.0008	
Max length of pores (μm) in the irregular part	V	H	DAS	Sr	Ti	Mg		Ts	
	*t*	5.91321	7.14815	4.55116	−5.5561	6.40432		3.22851	0.8169
	*p*	0.000000	0.00000	0.00001	0.000000	0.00000		0.001584	
	cte	1007.2	5.49	0.59	−1168.	5914.4		0.23	
Average length. of pores (μm) in the irregular part (µm)	V	H	DAS	Sr	Ti	Mg			
	*t*	5.32892	16.51022	3.49103	−5.0497	5.11986			0.7745
	*p*	0.000000	0.000000	0.00066	0.00000	0.00000			
	cte	573.04	4.378	0.283	−670.33	2984.83			
Density of pore length (#/mm^2^) in the exponential part	V	Int_HTi	DAS	Sr					
	*t*	4.41018	−2.9811	−3.9054					0.2430
	*p*	0.00002	0.003429	0.00015					
	cte	4159.8	−1.896	−0.742					

## Data Availability

Data will be made available upon request.
